# A comparative analysis of vaccine administration in urban and non-urban skilled nursing facilities

**DOI:** 10.1186/s12877-016-0320-4

**Published:** 2016-07-29

**Authors:** Yuan Pu, Veronika Dolar, Azad L. Gucwa

**Affiliations:** 1 Department of Biomedical Sciences, Long Island University at Post, Brookville, NY USA; 2 Department of Economics, Long Island University at Post, Brookville, NY USA

**Keywords:** Skilled nursing facilities, Influenza vaccine, Pneumococcal vaccine, Five-star quality ratings, Urban, Non-urban

## Abstract

**Background:**

The U.S. population is aging at an unprecedented rate, resulting in an increased demand for skilled nursing facilities (SNFs) and long-term care. Residents of these facilities are at a high risk for pneumococcal disease or severe influenza-related illnesses and death. For these reasons, the Centers for Medicare and Medicaid Services use influenza and pneumococcal vaccination rates as a quality measure in the assessment of SNFs, as complications related to these infections increase morbidity and mortality rates.

**Methods:**

Disparities have been reported amongst vaccination with increased rates in urban areas as compared to their non-urban counterparts. Statistical analyses were performed to compare influenza and pneumococcal vaccination in urban and non-urban SNFs to determine variables that may influence vaccination status.

**Results:**

Of the 15,639 nursing homes included in the study, 10,107 were in urban areas, while 5532 were considered non-urban. We found the percent of eligible and willing residents with up-to-date influenza and pneumococcal vaccinations increased with overall five-star ratings of SNFs. Somewhat paradoxically, although urban SNFs had higher mean overall five-star ratings, they showed lower rates of influenza and pneumococcal vaccination compared to non-urban SNFs. Ordinary least squares regression analysis comparing overall ratings, type of ownership, and geographic location by region yielded statistically significant results in which the overall rating, ownership-type and certificate-type favored urban SNFs (*p < 0.001*).

**Conclusions:**

This is the first systematic and comparative analysis to use the Nursing Home Compare database to assess vaccine administration of urban and non-urban SNFs. The findings of this study may be used to encourage the development of programs to improve vaccination rates and the quality of care in these facilities.

**Electronic supplementary material:**

The online version of this article (doi:10.1186/s12877-016-0320-4) contains supplementary material, which is available to authorized users.

## Background

Skilled nursing facilities (SNFs) provide continuous monitoring and medical assistance for individuals with long term healthcare needs. It is estimated that those aged 65 years and older are expected to comprise nearly 20 % of U.S. residents by 2050 [1]. This expansion will undoubtedly result in a concurrent increase in the demand for SNFs and long-term care to satisfy the needs of the aging population.

Since November 2002, consumers have been able to access information regarding SNFs from the Centers for Medicare and Medicaid Services (CMS). This database is updated periodically, and is available via the Nursing Home Compare website [2]. An overall Five-star Quality Rating System, determined by CMS, provides assessments of SNFs based on comprehensive ratings derived from health inspections ratings and adjusted staffing and quality measure ratings, offering a simplified way to assist consumers in making informed decisions [3]. It remains unclear as to whether this public database provides consumers with a better understanding of the facilities they are considering [4]. It has been found that consumers are more likely to consider matters of convenience rather than quality of clinical care received in SNFs [5]. In addition, a recent study found very little evidence to suggest that the introduction of the Nursing Home Quality Initiative (NHQI) report card measures led to increased consumer demand or better quality for long-stay stay residents [6]. Others have contradicted this, indicating that public reporting of these assessments resulted in little increase in consumers preferring higher-scoring facilities [7]. Nonetheless, these are good signs that consumers are beginning to use the information provided by CMS to choose the appropriate SNF for their care.

More than 15,000 SNFs are monitored by the CMS. Among them, over 60 % are located in urban areas. For reasons related to practices and costs, one would expect there to be some differences between urban and non-urban SNFs. It has previously been found that that rurality may be associated with poorer quality care [8]. When controlling for state and adjusting for SNF size and ownership, non-urban SNFs were less likely to earn a 4-star or higher quality rating. Additionally, not-for-profit and government-owned facilities usually earn higher ratings and provide a higher quality of care than for-profit SNFs on average [9]. SNF size and ownership are also important factors, and are relevant to non-urban SNFs health outcomes. Other studies have suggested that differences may exist also at access, quality, and cost in urban and non-urban SNFs [10, 11]. For these reasons, it has been suggested that efforts to enhance Medicare payment approaches, training programs for staff, facility accreditation status, as well as offering special care programs could likely reduce health disparities in non-urban SNFs [12].

Acute respiratory illnesses due to influenza or pneumococcal pneumonia are common in individuals residing in SNFs. Among Americans aged 65 years and older, influenza accounts for more than 190,000 hospitalizations and 33,000 deaths annually, and close to 3400 deaths due to pneumonia [13, 14]. The World Health Organization (WHO) has classified pneumococcal infections, caused by *Streptococcus pneumonia*, as a leading cause of morbidity and mortality worldwide [15]. It is approximated that 1.6 million people die every year from pneumococcal infections globally. Additionally, coinfection with *S. pneumoniae* and influenza has been observed to have a synergistic effect and increased mortality rates [16, 17]. Because individuals residing in SNFs are considered at high risk of developing complications, these diseases pose a major health concern as they are associated with increased morbidity, mortality and cost [18].

The Centers for Disease Control and Prevention (CDC) has recommended vaccine administration for adults aged 65 years of age and older, as well as for residents of SNFs and other long-term care facilities in an effort to reduce influenza- and pneumonia-related morbidity and mortality [19]. Although there has been some contention regarding the effectiveness of these vaccines particularly in the elderly, there is also evidence that vaccination is an important prevention strategy and significantly reduces hospitalization and related complications [20, 21]. Despite this debate, efforts are being made by some countries to increase vaccination rates. An example of this is in the United Kingdom, where clinical governance programs have successfully increased vaccinations through audits [22]. Canada has also suggested expanding its current vaccination program, and has recommended that it should include both residents and staff of SNFs reasoning that preventing transmission of influenza and other infectious agents within these facilities is relevant to quality of life [23].

The percent of long-stay residents assessed and appropriately given the seasonal influenza and pneumococcal vaccine are quality measures used in evaluating SNFs [24]. It is important to note that it is possible for this percentage to reach as high as 100 %. This is because the vaccination status of residents reported by SNFs do not represent vaccine administration rates, but rather include the total number of residents who had an up-to-date vaccine, those who were offered and declined the vaccine, as well as residents who were ineligible due to medical contraindications. A study investigating the actual proportion of residents vaccinated with either the influenza or pneumococcal vaccine were as low as 58.5 and 34.6 %, respectively [25]. Although an important quality indicator, vaccination rates are currently *not included* in the determination of the overall five-star ratings of SNFs [26].

Differences between urban and non-urban vaccination rates have previously been reported with lower rates in non-urban residents [27, 28]. It has been found that a multitude of factors affect administration of the seasonal influenza vaccine including healthcare-related social determinants such as knowledge and attitudes about vaccination and guidance from clinicians [27, 29]. Furthermore, individuals in non-urban locations are more dependent upon clinical settings for their influenza vaccinations [28]. The purpose of this study was to determine if a disparity exists in vaccine administration in SNFs located in urban versus non-urban areas and to investigate the relationship between vaccination rates and independent contributors.

## Methods

### Data source

A researchable database was created from two sources: 2013 CMS Nursing Home Compare datasets and 2010 Census Urban and Rural Classification and Urban Area Criteria for U.S. counties by state. These were the most recently updated reports at the time of this study.

### Sample

All U.S. Medicaid and Medicare SNFs were manually matched by geographic locale (urban, non-urban). The Census Bureau identifies Urbanized Areas (UAs) as 50,000 or more people and were used to determine categorize SNFs. “Rural” (non-urban), encompassed all population, housing, and territories not included within an urban area. The two databases used in this study did not report locales in the same format, spellings or abbreviations of county names. Therefore, facilities located in urban counties were determined and the remaining facilities were categorized as non-urban SNFs. There were 15,639 SNFs in the CMS Nursing Home Compare dataset; however, some had missing values so we were left with 15,509, of which 10,107 (65.2 %) were urban and 5532 (35.7 %) were non-urban.

SNFs were further separated by U.S. Regions: Northeast, Midwest, South, and West according to U.S. Census Bureau Regions and Divisions with State. Region 1 were the Northeast, New England and Middle Atlantic regions, which included 2640 (16.9 %) SNFs. Region 2 comprised the Midwest East, North Central and West North Central areas and included 5150 (32.9 %) SNFs. Region 3 was South, and included the South Atlantic, East South Central and West South Central regions with 5429 (34.7 %) SNFs. Region 4 was West and included the Mountain and Pacific regions and 2420 (15.5 %) SNFs.

All other classifications were based on provider information obtained from the CMS Nursing Home Compare dataset. Our customized database (Additional file 1) also classified SNFs by ownership-type and certificate-type. There were three types of ownership: for-profit, not-for-profit, and government-owned. Of the two certificate types, Medicare and Medicaid, SNFs qualified for either both Medicare and Medicaid (Category 0), Medicaid (Category 1) and Medicare (Category 2).

### Statistical analysis

We used non-parametric Wilcoxon rank-sum test (ranksum), also known as Mann-Whitney two-sample statistics, in order to compare mean overall rating of SNFs and rates of influenza and pneumococcal vaccination. A number of preliminary tests were performed in order to test for normality and equality of populations. Skewness and kurtosis test (sktest) and Shapiro-Wilk test (swilk) rejected the null hypothesis of normally distributed data samples. In addition, Kruskal-Wallis non parametric equality of population rank test (kwallis) for testing whether samples originate from the same distribution led us to reject the hypothesis that the populations are the same. As a result independent t-tests were deemed inappropriate.

Ordinary least squares (OLS) regression models were also performed using either influenza vaccination or pneumococcal vaccination as the dependent variable and urban locales as independent variables. In addition, OLS models controlled for overall rating, as well as for geographic locale, ownership type, and certificate type, since these covariates were all associated with vaccination.

All classifications were based on provider information sections of CMS nursing home data. For all statistical analyses, alpha was set at a *p* value less than *0.05*. Microsoft® Office Excel was used to create customized database in this study (Additional file 1). STATA/SE 13.1 was used to complete all statistical analyses performed for cases.

## Results

**Fig. 1:**
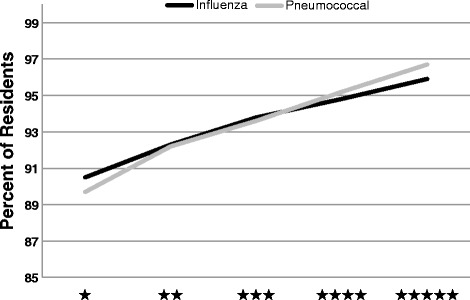
Mean percent of long-term residents assessed and appropriately given the seasonal influenza and pneumococcal vaccine increased with star value

Of the 15,639 SNFs included in the study, 10,107 were in urban areas, while 5532 were considered non-urban. The majority (64.6 %) of SNFs were located in urban areas. We found the mean percent of long-term residents assessed and appropriately given the seasonal influenza and pneumococcal vaccine increased with the overall star-rating of SNFs, suggesting that higher quality SNFs were more likely to vaccinate (Fig. 1).

**Fig. 2:**
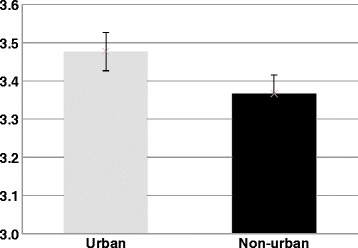
Mean five-star rating of urban (*n* = 10,107) and non-urban (*n* = 5532) SNFs. The difference in these means was determined to be significant (*p* < 0.001). Error bars indicate standard error mean

We also found the mean overall five-star ratings for urban SNFs were 3.48, and 3.37 for non-urban SNFs (Fig. 2). This difference was significant (*p < 0.001)* and consistent with previous studies [8]. Although we recognize this significance may be due to the large sample size, each star increase in actuality represented a 25 % increase because the lowest rating a facility could receive is 1. Accordingly, the difference in these means (0.11) was equivalent to a 2.75 percentage point increase, which is a more meaningful difference. In agreement with this, over 20 % of non-urban SNFs had five stars (the highest rating) as compared to approximately 30 % in urban areas (data not shown), suggesting urban SNFs received higher overall ratings on average.

**Fig. 3:**
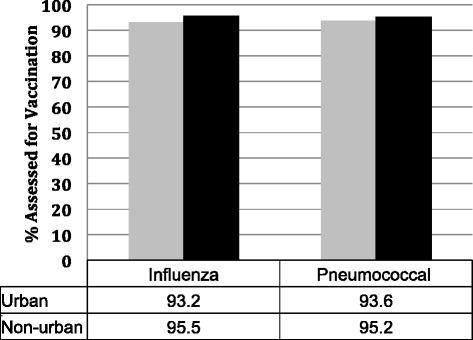
Proportion of influenza and pneumococcal vaccination in urban and non-urban skilled nursing facilities

Paradoxically, we found that although overall ratings were associated with increased vaccination rates (Fig. 1), urban SNFs showed lower rates of influenza and pneumococcal vaccination as compared to non-urban SNFs (*p < 0.00*1) (Fig. 3). Interestingly, in SNFs with higher ratings, pneumococcal assessment and vaccination was found to be higher than that of the seasonal influenza vaccine; however, this could possibly be because the influenza vaccine is administered yearly while the pneumococcal vaccine is only given once.

In order to further investigate the relationship between vaccination rates and urban locale, we performed ordinary least squares (OLS) regression analysis. We used either influenza vaccination (first two columns in Table 1) or pneumococcal vaccination (last two columns in Table 1) as the dependent variable and urban locales as independent variables. In addition, OLS models controlled for overall rating, as well as for geographic locale, ownership type, and certificate type, since these covariates were all associated with the rates of vaccination.

**Table 1:** Ordinary least squares (OLS) regression results estimating the effect of location, ownership-type and certificate-type on vaccination rates

	Influenza Vaccination		Pneumococcal Vaccination	
	Coef.	*p* value	Coef.	*p* value
Urban	−2.25	0.00**	−1.45	0.00**
Overall ratings	1.21	0.00**	1.56	0.00**
Geographic locale	−0.77	0.00**	−0.72	0.00**
Ownership-type	1.07	0.00**	1.12	0.00**
Certificate-type	0.42	0.03*	0.01	0.95

Significant results are marked with **(*p < 0.001*) or *(*p < 0.05*)

In this regression, the urban locale was again statistically significant and a negative sign in front of the coefficients (−2.25 for influenza and −1.45 for pneumococcal vaccination), suggesting that the rate of influenza vaccination were 2.25 percentage points lower for urban SNFs and 1.45 percentage points lower for pneumococcal vaccinations. These results hold even as we controlled for overall rating, geographic locale, type of ownership, and certificate type – variables that were all independently statistically significant in the regression. Moreover, this regression found that SNFs with only one certificate favored vaccination with the seasonal influenza vaccine compared to dual-eligible facilities. Using the Mann-Whitney test, there was a statistically significant difference (*p < 0.05)* between facilities with Medicaid or Medicare only; however, certificate type did not influence pneumococcal vaccination rates.

**Fig. 4:**
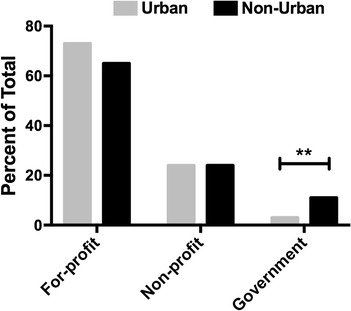
Proportion of skilled nursing facility ownership by location. Significant results are marked with ** (*p* < 0.001)

Finally, more than 65 % of SNFs included in this study were for-profit, and less than 10 % were owned by government amongst all SNFs (Fig. 4). In addition, there was a higher proportion of SNFs located in non-urban that were government-owned (chi-squared test, *p < 0.001*). Thus, non-urban SNFs were more likely to be government-owned and typically had higher rates of vaccination. Although not statistically significant, urban SNFs were also more likely to be for-profit, and less likely to vaccinate.

## Discussion

As quality measurements, vaccination rates are rarely studied. In this analysis, we showed that seasonal influenza and pneumococcal assessment and vaccination should be considered in the assessment of quality of care received in SNFs. Both urban and non-urban SNF influenza and pneumococcal vaccination rates increased relative to the overall five-star ratings. In addition, we found that overall ratings favored urban SNFs. Because urban SNFs were higher rated, one would assume they would in turn have higher vaccination rates; however, our data suggests the opposite. With access to vaccinations no longer a barrier within this clinical setting, it is possible the proportion of those vaccinated in SNFs is increased in comparison to the general public in non-urban areas [28].

Next, we wanted to explore factors that could potentially explain these results. We analyzed possible contributors to quality of SNFs as well as factors that could potentially influence vaccination rates using data provided by CMS. Ordinary least squares regression analysis considered the relationship between vaccinations with ownership-type, geographic location, and certificates in urban and non-urban settings. We found that ownership seemed to play an important role in this process. Government and non-profit SNFs favored vaccination. One possible explanation for this finding is that government-owned facilities may have stricter policies for vaccination assessment and administration processes. Because non-urban SNFs had more government-owned SNFs, this also may have impacted our finding that non-urban SNFs had higher influenza and pneumococcal vaccination rates but lower overall five-star ratings.

Another factor we controlled for was geographic locations. The Northeast SNFs had significantly higher vaccine rates while the South and the West seemed less likely to vaccinate their residents (data not shown). Weather conditions in the North may facilitate respiratory illnesses related to influenza and pneumococcal pneumonia. Population density may be another reason, as people living outside the Northern region may have fewer vaccinations. It is possible that racial issues may also interfere with our findings, as it has been found that individuals living in the South and West typically do not want to share their vaccination history [27].

Certificate-type plays a role in the financing of SNFs. Dual-eligible SNFs with certifications for both Medicare and Medicaid showed they qualify for services, perhaps even for some special procedures, which may directly reflect on their overall ratings. A study using a detailed national database of hospitalized patients discharged to SNFs found that dual-eligible patients from the same hospital were discharged to SNFs with worse quality of care compared to their Medicare-only counterparts [30]. Our analysis showed that SNFs with only one certificate had higher rates of the seasonal influenza but not the pneumococcal vaccine. Interestingly, we also found the Medicaid-only SNFs had higher rates (*p < 0.05*), Medicaid is a social health care program for families and individuals with low incomes. Because vaccines are recommended for individuals aged 65 and older, SNFs with a Medicare certificate would be expected to have higher rates.

We also found that non-urban SNFs were more often owned by the government compared to urban SNFs, which also typically vaccinate more often. In addition, urban facilities were more often for-profit and are also less likely to vaccinate. One could claim that better SNFs would vaccinate more as indicated more; however, the for-profit owned facilities may not have enough incentive to vaccinate. Likewise, if the overall rating system typically and accurately corresponds to better quality SNFs, then the current rating system does not seem to be adequately rewarding non-urban SNFs for their efforts. Alternatively, it would be interesting to investigate differences in vaccination practice and conventions between urban and non-urban facilities. It has been shown that publicly reported quality measures can affect performance, and that SNFs with objectives that required considerable improvement of quality measures resulted in more favorable outcomes [31].

It should be noted that there were several limitations to this study. First, overall quality of SNFs is determined from many different measures. The Nursing Home Compare database includes only some of the possible factors that could be considered when assessing a characteristic such as quality of clinical care. Patient and family satisfaction and participation should also be considered and included in the data analyzed for a study such as this.

A second limitation was that the data used in this study depends to a certain extent on how well SNFs complete the minimum data set that is used to construct the Nursing Home Compare quality measures. These measures are self-reported and it is possible that some variation in ratings may be attributable to how a SNF reports data rather than actual performance. Without confirmation of these data, self-reported assessments may not be entirely accurate because they are directly related to the overall ratings of these facilities.

Finally, we used the 2010 Census Urban and Rural Classification database in this study. There is also the U.S. Department of Agriculture County Continuum Codes, which we did not use to define as urban and non-urban locations in this study. Thus, urban and non-urban definitions may slightly differ based on function and population. The data used in this study may not accurately represent urban and non-urban locations in 2013.

## Conclusions

This is the first systematic and comparative analysis to use the Nursing Home Compare database to assess differences in vaccine administration in urban and non-urban SNFs. Ordinary least squares analysis indicated urban SNFs had a lower percent of eligible and willing residents with an up-to-date influenza and pneumococcal vaccination despite having higher overall star ratings. Our results suggest that among all variables investigated, vaccination rates were dependent on ownership-type in non-urban SNFs. In non-urban areas, there were more government-owned SNFs. It has previously been reported that consumers are most likely to make decisions regarding SNFs due to matters of convenience rather than the quality of clinical care. The results of this study can be used to encourage the development of programs to improve vaccination rates and the quality of care in these SNFs. Further research is needed to fully understand the process of vaccine administration in SNFs and how they influence the decision making process of consumers.

## Abbreviations

CDC, Centers for Disease Control and Prevention; CMS, Centers for Medicare and Medicaid Services; NHQI, Nursing Home Quality Initiative; SNF, skilled nursing facilities; WHO, World Health Organization

## Additional file

Additional file 1: Customized database used in this study. (XLSX 2173 kb)
